# A Systematic Approach for Multidimensional, Closed-Form Analytic Modeling: Effective Intrinsic Carrier Concentrations in *Ga*_1−x_*Al*_x_*As* Heterostructures

**DOI:** 10.6028/jres.107.008

**Published:** 2002-02-01

**Authors:** Herbert S. Bennett, James J. Filliben

**Affiliations:** National Institute of Standards and Technology, Gaithersburg, MD 20899-0001

**Keywords:** analytical expression, effective intrinsic carrier concentrations, gallium aluminum arsenide, heterostructures

## Abstract

A critical issue identified in both the technology roadmap from the Optoelectronics Industry Development Association and the roadmaps from the National Electronics Manufacturing Initiative, Inc. is the need for predictive computer simulations of processes, devices, and circuits. The goal of this paper is to respond to this need by representing the extensive amounts of theoretical data for transport properties in the multi-dimensional space of mole fractions of AlAs in Ga_1−_*_x_*Al*_x_*As, dopant densities, and carrier densities in terms of closed form analytic expressions. Representing such data in terms of closed-form analytic expressions is a significant challenge that arises in developing computationally efficient simulations of microelectronic and optoelectronic devices. In this paper, we present a methodology to achieve the above goal for a class of numerical data in the bounded two-dimensional space of mole fraction of AlAs and dopant density. We then apply this methodology to obtain closed-form analytic expressions for the effective intrinsic carrier concentrations at 300 K in n-type and p-type Ga_1−_*_x_*Al*_x_*As as functions of the mole fraction *x* of AlAs between 0.0 and 0.3. In these calculations, the donor density *N*_D_ for n-type material varies between 10^16^ cm^−3^ and 10^19^ cm^−3^ and the acceptor density *N*_A_ for p-type materials varies between 10^16^ cm^−3^ and 10^20^ cm^−3^. We find that p-type Ga_1−_*_x_*Al*_x_*As presents much greater challenges for obtaining acceptable analytic fits whenever acceptor densities are sufficiently near the Mott transition because of increased scatter in the numerical computer results for solutions to the theoretical equations. The Mott transition region in p-type Ga_1−_*_x_*Al*_x_*As is of technological significance for mobile wireless communications systems. This methodology and its associated principles, strategies, regression analyses, and graphics are expected to be applicable to other problems beyond the specific case of effective intrinsic carrier concentrations such as interpreting scanning capacitance microscopy data to obtain two-dimensional doping profiles.

## 1. Introduction and Motivation

Optoelectronic, microwave, and electronic devices made from III-V compounds have high carrier and/or doping concentrations in their active regions during operation. Such high concentrations produce changes in carrier densities of states, band structures, and effective intrinsic carrier concentrations. These changes then influence considerably the performance of optical and electronic devices at 300 K in advanced applications such as microwave receivers and transmitters, light sources, and high-speed digital electronics for signal processing, computing, and wide-band communications. The properties of the above devices that are altered by high-concentration effects include carrier mobilities, band gaps, bandedge offsets at heterostructure interfaces, densities of initial and final states, effective intrinsic carrier concentrations, refractive indices, absorption, and luminescence. Examples of devices for which the results from this paper are significant include linear power amplifiers in digital cellular phones and diode lasers and light emitting diodes in optical communications systems.

A critical issue identified in both the technology roadmap from the Optoelectronics Industry Development Association (OIDA) [1] and the roadmaps from the National Electronics Manufacturing Initiative (NEMI) [2] is the need for commercial simulators of processes, devices, and circuits that are predictive. Predictive simulators are key to being able to evaluate quickly manufacturing options and thereby to shorten product development cycles. A critical condition for predictive simulators is that they require physically and chemically correct models for input parameters. Predictive device simulators for bipolar and field-effect transistors require a variety of physical models and associated input parameters to describe fully how carrier transport varies with carrier concentrations, ionized dopant densities, alloy mole fractions, and temperatures. The effective intrinsic carrier concentration is an essential input parameter for bipolar device simulators. The goal of this paper is to respond to this need by representing the great amounts of numerical data for the effective intrinsic carrier concentrations in Ga_1−_*_x_*Al*_x_*As, which are presently in tables [3], in terms of closed-form analytic expressions. These expressions are given below. Combining these expressions and the recently reported minority electron mobilities [4] then gives an internally self-consistent description of carrier transport in the p-type bases of GaAs/Ga_1−_*_x_*Al*_x_*As heterojunction bipolar transistors (HBTs). This new, self-consistent description of carrier transport is based on quantum mechanics with no fitting parameters extracted from interpreting electrical measurements on the devices themselves. It will reduce the number of unknown or variational parameters in device simulators and should lead to improved predictive capabilities for device simulators.

The predictions of dc common-emitter gains, RF power gains, and current-voltage characteristics from simulators for silicon and III-V semiconductor bipolar transistors are sensitive to the dependence of the effective intrinsic carrier concentration *n*_ie_(*N*_I_, *x*) on the dopant density *N*_I_, where I = D for donors, I = A for acceptors, and *x* is the mole fraction of AlAs. But, because neither theoretical nor experimental data on the variation of *n*_ie_(*N*_I_, *x*) in Ga_1−_*_x_*Al*_x_*As with *N*_I_ and *x* are known very well, device simulators for GaAs/Ga_1−_*_x_*Al*_x_*As HBTs usually contain the physically questionable assertion that *n*_ie_(*N*_I_, *x*) = *n*_i_, where *n*_i_ is the intrinsic carrier concentration in the limits that both *N*_I_ and *x* approach zero.

For the reasons cited in the previous paragraph, this paper focuses on the model for how the effective intrinsic carrier concentrations vary with dopant density and mole fraction of AlAs in Ga_1−_*_x_*Al*_x_*As at 300 K. Self-consistent numerical solutions to the quantum mechanical, non-linear integral-differential equations for carrier transport in semiconductors result in discrete data points that by themselves do not readily suggest closed-form analytic expressions for carrier densities of states, band structure changes, and thereby, effective intrinsic carrier concentrations. Interpolating among the discrete data points in “look-up” tables leads to discontinuities, particularly when numerical differences must be used to compute first and higher order derivatives, and, as mentioned above, is computationally inefficient. Due to these computational inefficiencies, industry is reluctant to incorporate “look-up” tables in semiconductor device simulators that run on engineering workstations.

The motivation for our performing the following analyses is to derive closed-form analytic expressions that will result in more efficient computer simulations and improved insights on how the many physical mechanisms, which influence densities of states and band structures in ternary compound semiconductors and heterostructure devices, affect their electronic and optical behavior. Our data analyses, presented in the following sections, enable us to reduce the number of unknown parameters in numerical simulations that predict electrical and optical performance of devices such as bipolar transistors, solar cells, laser diodes, and light-emitting diodes.

**Fig. 1:**
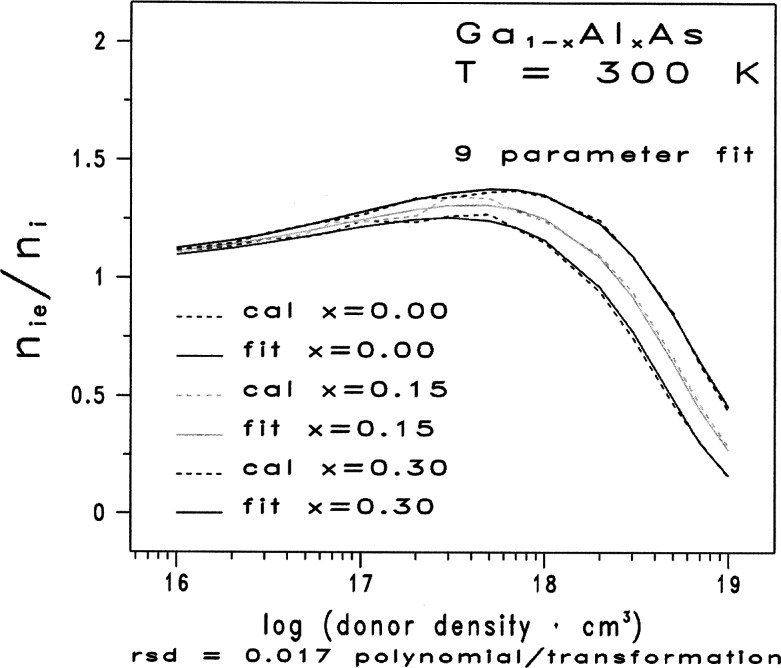
Normalized intrinsic carrier concentration ratios *n*_ie_/*n*_i_ for n-type Ga_1−_*_x_*Al*_x_*As as functions of the donor density for three representative values of mole fraction of AlAs. The three dashed curves show results from quantum mechanical calculations based on the Klauder fifth level of approximation for Ga_1−_*_x_*Al*_x_*As with *x* = 0.0, 0.15, and 0.30. The three solid curves show the closed-form, analytic fits based on Eq. (10). H. S. Bennett, High Dopant and Carrier Concentration Effects in Gallium Aluminum Arsenide: Densities of States and Effective Intrinsic Carrier Concentrations, J. Appl. Phys. **83**, 3102 (1998).

**Fig. 2:**
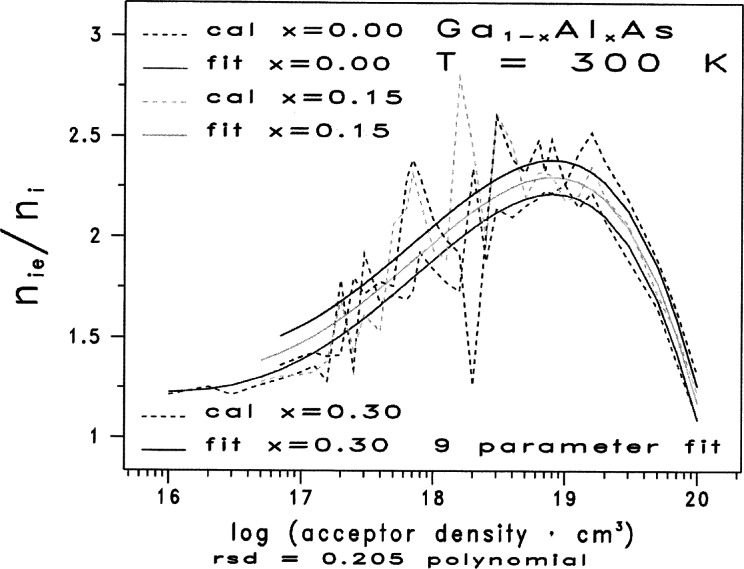
Normalized effective intrinsic carrier concentration ratios *n*_ie_/*n*_i_ for p-type Ga_1−_*_x_*Al*_x_*As as functions of the donor density for three representative values of mole fraction of AlAs. The three dashed curves show results from quantum mechanical calculations based on the Klauder fifth level of approximation for Ga_1−_*_x_*Al*_x_*As with *x* = 0.0, 0.15, and 0.30. The three solid curves show the closed-form, analytic fits based on Eqs. (11) and (12). H. S. Bennett, High Dopant and Carrier Concentration Effects in Gallium Aluminum Arsenide: Densities of States and Effective Intrinsic Carrier Concentrations, J. Appl. Phys. **83**, 3102 (1998).

The families of curves given in Figs. 1 and 2 represent graphically the two-dimensional, numerical tables consisting of discrete data points from the calculations reported in Ref. [3]. Such graphical representations are a common recourse when several complex and competing physical mechanisms occur and when multidimensional, closed-form analytic expressions are not available. Incorporating such discrete data points into physical models for use in computer simulations is usually not satisfactory due to excessive CPU time associated with interpolations between the discrete data points.

We apply in this paper the general strategy given in Ref. [4] for obtaining closed-form analytic expressions from multi-dimensional tabular data to the multi-dimensional tabular data in Tables 1 and 2 for effective intrinsic carrier concentrations. That general strategy was based on separable functions, melding functions, transformations, admissible non-linear methods, and regression analyses to obtain multi-dimensional, closed-form analytic expressions.

To obtain acceptable analytic fits to the discrete theoretical values for the effective intrinsic carrier concentrations in semiconductor device simulators that run on engineering workstations, we want to have relative residual standard deviations for the analytic fits that are reasonably small, i.e., usually less than 2 %. And we want to achieve such residual standard deviations with as small a number of fitting parameters compared to the total number of data points as possible.

The development of such analytic fits would represent a significant increase in computational efficiency by about a factor of 5 and would give analytic expressions for the normalized effective intrinsic carrier concentrations for use in commercial semiconductor device simulators that are in much closer agreement with known device physics than the expressions currently used. The combination of the existing NIST supercomputer-generated data for normalized effective intrinsic carrier concentrations and the derived two-dimensional analytic fits will lead to computer simulators that are at once both more parsimonious (have fewer unknown or tuning-variational parameters) and more accurate (offer improved predictability).

## 2. Effective Intrinsic Carrier Concentrations

The methodology from Ref. [3] of the quantum mechanical calculations on which this paper is based is summarized here as a way to explain notation and for completeness. We end this section with a discussion of possible sources for the scatter in the computed results near the Mott transition.

Equations (2), (3), and (6) that are given below for the electron density, hole density, and the screening radius, respectively, all depend on knowing the densities of states for the carriers. Reference [3] contains detailed discussions on how the integral equations for the densities of states in the valence and conduction bands are solved. The solutions involve the discretization of quantum mechanical integral equations that yield large sets of complex algebraic equations. The complex algebraic equations are then cast into large matrix equations. After discretization, the integral equations may be expressed in terms of matrix equations like the following:

$$\sum_{J=1}^{J_{\max}}C\left(I,J\right)X\left(J\right)=B\left(I\right),$$ (1)

where ***X***(*J*) represents renormalized self-energy factors, ***B***(*I*) represents the inhomogeneous term that is proportional to the Fourier transform of the scattering potential, ***C***(*I*, *J*) = ***A***(*I*, *J*) + ***I*** is a complex matrix, ***I*** is the identity matrix, and ***A***(*I*, *J*) represents the two-di-mensional integrands involving renormalized Green’s functions. The dimension of array ***X*** is *J*_max_, and *J*_max_ equals the product of the number of values of wave numbers *N*_kmax_ times the number of values of angles *N*_*μ*max_ used in performing numerical integrations.

For the case in which *N*_kmax_ = 42, *N*_μmax_ = 8, and *J*_max_ = 336, it takes about 85 h of Cray^1^ computer time to calculate *n*_ie_(*N*_1_) for one value of *x* and 35 values of dopant densities *N*_1_. About 97 % of the total CPU time is spent in one library subroutine that factors the complex matrix ***A***(*I*, *J*) by Gaussian elimination and estimates its condition in preparation for the next subroutine that solves the complex system given by Eq. (1) for **X**(*J*), the discrete representation of the self-energy factors.

The electron *n* and hole *p* concentrations at thermal equilibrium are given, respectively, by

$$n=\int_{-\infty }^{+\infty }f_{0}\left(E\right)\;\rho_{c}\left(E\right)\;dE$$ (2)

and

$$p=\int_{-\infty }^{+\infty }\left[1-f_{0}\left(E\right)\right]\;\rho_{v}\left(E\right)\;dE,$$ (3)

where *ρ*_c_ is the density of states for the conduction band, *ρ*_v_ is the density of states for the valence band, *E* is the carrier energy,

$$f_{0}\left(E\right)={\left[1+\exp\left\{E-E_{F})/k_{B}T\right\}\right]}^{-1}$$ (4)

is the Fermi-Dirac distribution function, *E*_F_ is the Fermi energy, *k*_B_ is the Boltzmann constant, and *T* is the thermodynamic temperature in kelvins. Because the carrier-carrier interactions that give rise to exchange and correlation energies become significant at high concentrations, the calculations of *n* and *p* from Eqs. (2) and (3) require estimates for these carrier-carrier interactions. In terms of the above quantities, the effective intrinsic carrier concentration is given by

$$n_{\mathrm{ie}}={\left(n\;p\right)}^{1/2}.$$ (5)

The quantum mechanical calculations incorporate the Thomas-Fermi expression for the screening radius,

$$r_{s}^{2}=-\frac{4\pi e^{2}}{\kappa }\int_{-\infty }^{+\infty }\frac{df_{0}\left(E\right)}{dE}\left[\rho_{c}\left(E\right)-\rho_{v}\left(E\right)\right]dE,$$ (6)

and the charge neutrality condition,

$$N_{I}=n-p,$$ (7)

to compute self-consistently the Fermi energy *E*_F_ and the Thomas-Fermi screening radius *r*_s_ for given ionized dopant concentration *N*_I_ and temperature *T*. The static dielectric constant is denoted by *κ* in dimensionless units. The ionized dopant concentration is positive for n-type material (donor ions) and negative for p-type material (acceptor ions). The results reported here are for uncompensated material.

We also use the normalized effective intrinsic carrier concentration; namely,

$$n_{\mathrm{ie}}/n_{i}={\left(n\;p\right)}^{1/2}/{\left(n_{0}\;p_{0}\right)}^{1/2}.$$ (8)

where the intrinsic carrier concentration *n*_i_ =lim_*N*I→_o*n*_ie_=(*n*_0_p_0_)^1/2^.

### 2.1 n-Type Ga_1−_*_x_*Al*_x_*As

The dashed curves in Fig. 1 represent the results calculated from the methods presented in Ref. [3] and give the normalized effective intrinsic carrier concentration *n*_ie_/*n*_i_ for n-type Ga_1−_*_x_*Al*_x_*As at 300 K. They are based on evaluating *n*_ie_/*n*_i_ for 16 values of *N*_D_ between 10^16^ cm^−3^ and 10^19^ cm^−3^ and for three values of the mole fraction of AlAs, *x* = 0.00, 0.15, and 0.30.

### 2.2 p-Type Ga_1−_*_x_*Al*_x_*As

The dashed curves in Fig. 2 represent the results calculated from the methods presented in Ref. [3] and give the normalized effective intrinsic carrier concentration ratios *n*_ie_/*n*_i_ for p-type Ga_1−_*_x_*Al*_x_*As at 300 K for 35 values of *N*_A_ between 10^16^ cm^−3^ and 10^20^ cm^−3^ and for three values of the mole fraction of AlAs *x* = 0.00, 0.15, and 0.30. The conclusions for p-type Ga_1−_*_x_*Al*_x_*As are qualitatively similar to those for n-type Ga_1−_*_x_*Al*_x_*As whenever acceptor densities are sufficiently far from the Mott transition. But considerable scatter in the calculated *n*_ie_/*n*_i_ values occurs for the mid-range of acceptor densities that spans the Mott transition, particularly for the decade of acceptor densities from 10^18^ cm^−3^ to 10^19^ cm^−3^. In this paper, we define the Mott transition as that doping density for which the screened Coulomb potential no longer has bound states.

The Mott transition for n-type Ga_1−_*_x_*Al*_x_*As occurs for a range of doping densities that is of much less technological interest. This is fortunate when computing high concentration effects in n-type Ga_1−_*_x_*Al*_x_*As. However, the Mott transition for p-type Ga_1−_*_x_*Al*_x_*As occurs near doping densities of technological significance. For example, the Mott transition in p-type Ga_1−_*_x_*Al*_x_*As occurs near acceptor densities typically used in the bases of HBTs for the linear power amplifiers in the front ends of microwave and millimeter wave receivers.

The ratios of *n*_ie_/*n*_i_ are evaluated at 23 acceptor densities for p-type Ga_1−_*_x_*Al*_x_*As between 10^17^ cm^−3^ and 10^19^ cm^−3^. This number is more than twice the number of evaluation points over the same two decades given in Fig. 1 for n-type Ga_1−_*_x_*Al*_x_*As. The ratios of *n*_ie_/*n*_i_ are evaluated at 12 values of acceptor density for the decade between 10^18^ cm^−3^ and 10^19^ cm^−3^. The observed numerical scatter for the values of *n*_ie_/*n*_i_ in p-type Ga_1−_*_x_*Al*_x_*As tend to vary from about 4 % away from the Mott transition to about 20 % near the Mott transition.

The scatter of *n*_ie_/*n*_i_ values in Fig. 2 for the mid-range of acceptor densities that spans the Mott transition arises from a combination of six effects:

1. adaptive grid spacings used in the evaluation of the two dimensional integrals over wave numbers and over angles;
2. algorithm used to solve the very nonlinear matrix equations in Ref. [3];
3. method used to locate the band-edges;
4. method used to determine when the distorted, Klauder densities of states at sufficiently high energies are asymptotic to the undistorted, parabolic densities of states;
5. method used to determine when the carrier quantum mechanical states are spatially compact (localized) and when they are spatially extended (conducting); and
6. physics of the Mott transition with bifurcation of bound and continuum states.

Additional theoretical, computational, and experimental research is needed to determine which of the foregoing six effects dominate. Separating the physics effects (item 6 above) from the numerical effects (items 1 to 5 above) is not possible at present. The authors of Ref. [5] use interpolation and extrapolation methods to obtain the variation of *n*_ie_^2^/*n*_i_^2^ ie i with acceptor density (Fig. 3 of Ref. [5]) from their experimental measurements for *n*^2^ie *D*min and *D*_min_, where *D*_min_ is the minority carrier diffusivity. They report therein five values of acceptor densities over the same decade from 10^18^ cm^−3^ to 10^19^ cm^−3^ and obtain less scatter in the experimentally extracted data with a larger grid spacing for the dopant density than the scatter in the theoretically calculated data with a much smaller grid spacing for the dopant density, which is reported here.

Attempts to reduce the scatter by decreasing the integration grid spacings further so that *N*_kmax_ = 842, *N*_μmax_ = 15, and *J*_max_ = 12630, and resulted in failures of the numerical solutions to converge after unacceptable, excessive CRAY CPU times. Also, when numerical solutions for Eq. (8) were attempted on clusters of multiprocessor, high-end workstations, similar results occurred.

Because such scatter as shown in Fig. 2 in the vicinity of the Mott transition is not acceptable for “look-up tables” in device simulators and because the limited experimental data suggest a reasonably smooth behavior in this region, we exercise care in our nonlinear statistical analyses to avoid fitting the scatter. But, we should always keep in mind that, even though present experimental data may suggest reasonably smooth behavior of *n*_ie_ through the Mott transition region, future improved theoretical and experimental data could reveal additional structure in the dependence of *n*_ie_ on the dopant density and mole fraction in the vicinity of the Mott transition.

## 3. Data Table for Effective Intrinsic Carrier Concentrations in n-Type Ga_1−_*_x_*Al*_x_*As

This section describes the background details by which the data in Table 1 for the n-type normalized effective intrinsic carrier concentrations were obtained. This data serves as our starting point for deriving the closed-form analytic expression for the normalized effective intrinsic carrier concentration in n-type Ga_1−_*_x_*Al*_x_*As.

The theoretical calculations in Ref. [3] were done for a full factorial design consisting of 16 discrete values of donor density *N*_D_ between 10^16^ cm^−3^ and 10^19^ cm^−3^ and 3 discrete values of mole fraction *x* between 0.0 and 0.30, namely, *x* = 0.0, 0.15, and 0.30 [denoted also by *x* = 0.00 (0.15) 0.30], to yield a total of 48 data points. We also use the notation that *x*_1_ = 0.00, *x*_2_ = 0.15, and *x*_3_ = 0.30. The self-consistent, numerical solutions to the quantum mechanical, non-linear integral-differential equations for *n*_ie_ are given in Table 1 as a two-dimensional array of discrete data points. This data representation, as opposed to a functional representation, was necessary because the several competing physical mechanisms do not readily yield any acceptable theoretical closed-form analytic expression.

The 48 data points, presented in Table 1, are represented graphically in Fig. 1 as a family of three dashed traces corresponding to the three mole fraction values, *x* = 0.00 (0.15) 0.30, respectively. The fixed increment of *x_i_*−*x_i_*_−1_ = 0.15 for all *i* and a subsequent fortuitous response surface in the mole-fraction variable will be advantageously employed later to simplify the fitting process.

We thus have the task of finding a closed-form, two-dimensional analytic function $Y_{n}^{f}(X,x)$ for the normalized effective intrinsic carrier concentration in n-type Ga_1−_*x*Al*_x_*As, such that $Y_{n}^{f}(X,x)$ is a good fit to $Y_{n}^{f}(X,x)\approx n_{\mathrm{ie}}$(n-type; *N*_D_, *x*)/*n*_i_, where *X* = log_10_(*N*_D_/10^16^ cm^−3^).

## 4. Data Analysis and Final Results for *n*_ie_/*n*_i_ in n-Type Ga_1−_*_x_*Al*_x_*As

We show that using a combination of separable functions, transformations on the discrete data points in Fig. 1, and non-linear regression analyses lead to a single two-dimensional, closed-form, analytic expression for the normalized effective intrinsic carrier concentration at 300 K in n-type Ga_1−_*_x_*Al*_x_*As as a function of the mole fraction *x* of AlAs between 0.0 and 0.3 and the donor density *N*_D_ between 10^16^ cm^−3^ and 10^19^ cm^−3^. Throughout our analyses, we rely substantially on graphics and keep the number of fitting coefficients to a minimum, subject to the constraint that the residual standard deviation, *S*_res_(*Y^f^*), in the original units satisfies *S*_res_(*Y^f^*) ≤0.02. The residual standard deviation is a measure of the “average” error in a fitted model and thereby is a metric for assessing the quality of the fit. A smaller *S*_res_(*Y^f^*) indicates a better fit. The residual standard deviation for a model *Y^f^* = *f*(*X*, *x*), is

$$S_{\mathrm{res}}\left(Y^{f}\right)=\sqrt{\left[\sum_{j=1}^{N}Y_{j}-{\bar{Y}}_{j}^{f}{)}^{2}/\left(N-P\right)\right],}$$ (9)

where *Y_j_* are the observed data values, the ${\bar{Y}}_{j}^{f}$ are the predicted values from the fitted model, *N* is the total number of data points (here *N* = 48), and *P* is the total number of parameters to be fitted in the model.

We use the NIST-developed DATAPLOT [6] software for both the exploratory graphics and for the extensive non-linear statistical analyses. Also, for those cases in which the residual standard deviations from analyses based on different functional forms are quantitatively similar, we select the functional form that will minimize the CPU time when the closed-form analytical function is used in commercial simulators and select procedures that have a minimum of fitting parameters.

Our general strategy here is based on separable functions and on transformations of the response function *Y*_n_ that give near-linear separable functions as described below. We want to obtain the function $Y_{n}^{f}=f(X,x)$ in the two-dimensional continuum space spanned by *X* = log(*N_D_*/10^16^ cm^−3^) and *x*. This bounded two-dimensional continuum is given in Fig. 1 with 0≤ *X* ≤3 and 0.00 ≤ *x* ≤ 0.30. As with fitted functions, extreme caution must be exercised in extrapolating beyond these *X* and *x* limits.

We consider the discrete two-dimensional space given by the 48 data points in Fig. 1 and in Table 1. Using the methodology in Ref. [4], we find that an acceptable fit, which meets the conditions on residual standard deviation and number of fitting parameters, is obtained by the six steps that follow. Steps 1 to 4 are the essential steps that remain after completing exploratory graphics on the 48 discrete points of the data set.

1. Transform the data *Y*_n_ = *f*(*X*, *x*) in Table 1 to the natural logarithmic space,
  *y*_n_(*X*, *x*) = ln[*Y*_n_(*X*, *x*)].
2. Choose the *x* = 0.0 GaAs data for *y*_n_(*X*, 0) as the base or reference function and fit *y*_n_(*X*, 0) to the quartic function *A*(*X*), where
  *A*(*X*) = *a*_0_ + *a*_1_*X* + *a*_2_*X*^2^ + *a*_3_*X*^3^ + *a*_4_*X*^4^
  This gives values for the fitting parameters *a*_i_ that will be used as starting values in later steps.
3. Fit the 2 sets of differencesδ_i_(*X*) = *y*_n_(*X*, *x*_i_) − *y*_n_(*X*, 0) to Lorentzians *L*_i_(*X*), where
  *L*_i_(*X*) = *m*_i_/[1 + {(*X c*_i_)/*d*_i_}^2^ ].
4. Because the fitting parameters *c*_i_ and *d*_i_ are essentially independent of the mole fraction, we then fit the combined sets of differences to the Lorentzian *B*(*X*, *x*), where
  *B*(*X*, *x*) = (*b*_0_ + *b*_1_ *x*)/[1 + {(*X*−c))/*d*}^2^]
  Again, this gives values of additional fitting parameters for later use.
5. Using the fitting parameters from steps 1 to 4 above as starting values, fit *y*_n_(*X*, *x*) to the function *y*_n_*^f^* (*X*, *x*) = *A*(*X*) + *B*(*X*, *x*)
6. Using the fitting parameters from step 5 as starting values, fit *Y*_n_(*X*, *x*) to the function

$$Y_{n}^{f}\left(X,x\right)=\exp\left[A\left(X\right)+B\left(X,x\right)\right].$$ (10)

In summary, Eq. (10) for $Y_{n}^{f}(X,x)$ is the two-dimensional, closed form analytic expression for the data set *Y*_n_(*X*, *x*) containing 48 discrete data points. The nine fitting parameters for the normalized effective intrinsic carrier concentration for n-type Ga_1−_*_x_*Al*_x_*As, *Y*_n_ = *n*_ie_ (n-type; *N*_D_, *x*)/*n*_i_ from Eq. (10), are given in Table 3, where the donor density is *N*_D_, the mole fraction of AlAs is *x*, and the intrinsic carrier concentration is *n*_i_. All of the fitting parameters are dimensionless. The residual standard deviation is *S*_res_(*Y*) = 0.017. The other expressions in Eq. (10) are:

$$\begin{array}{l} A\left(X\right)=a_{0}+a_{1}\;X+a_{2}\;X^{2}+a_{3}\;X^{3}+a_{4}\;X^{4},\\ B\left(X,x\right)=\left(b_{0}+b_{1}\;x\right)/\left[1+{\left\{\left(X-c\right)/d\right\}}^{2}\right],\;\text{and}\\ \;X=\log_{10}\left(N_{D}/10^{16}{\mathrm{cm}}^{-3}\right). \end{array}$$

The analytic expression in Eq. (10) now enables quantum mechanically based results, which required tens of hours of supercomputer time, to be readily and efficiently incorporated into commercial, workstation-based simulations of Ga_1−_*_x_*Al*_x_*As devices. However, the analytic fit in Eq. (10) is valid only within the bounded space of 0 ≤ *X* ≤ 3 and 0 ≤ *x* ≤ 0.30, and must not be used beyond this bounded two-dimensional space in which it is derived.

## 5. Data Table for Effective Intrinsic Carrier Concentrations in p-Type Ga_1−_*_x_*Al*_x_*As

This section describes the background details by which the data in Table 2 for the p-type normalized effective intrinsic carrier concentrations were obtained. As before in the case of n-type Ga_1−_*_x_*Al*_x_*As, this data is the starting point for deriving the closed-form analytic expression for the normalized effective intrinsic carrier concentration in p-type Ga_1−_*_x_*Al*_x_*As.

The theoretical calculations in Ref. [3] were done for a full factorial design consisting of 35 discrete values of acceptor density *N*_A_ between 10^16^ cm^−3^ and 10^20^ cm^−3^ and three discrete values of mole fraction *x* between 0.0 and 0.30, namely, *x* = 0.0, 0.15, and 0.30, to yield a total of 98 data points. The seven entries denoted by blanks in Table 2 for some *N*_A_ values between 10^16^ cm^−3^ and 5 × 10^16^ cm^−3^ when *x* = 0.15 or *x* = 0.30 mean that excessive CPU time would have been required for convergence.

The 98 data points in Table 2 are represented graphically in Fig. 2 as a family of three dashed traces corresponding to the three mole fraction values *x* = 0.00 (0.15) 0.30, respectively. Again, the fixed increment of *x_i_*−*x_i_*_−1_ = 0.15 for all *i* and a subsequent fortuitous response surface in the mole-fraction variable will be advantageously employed later to simplify the fitting process.

We now have the task to find a closed-form, two-dimensional analytic function ${Y_{p}}^{f}$ for the normalized effective intrinsic carrier concentration in p-type Ga_1−_*_x_*Al*_x_*As, such that ${Y_{p}}^{f}(X,x)\approx n_{\mathrm{ie}}$ (p-type; *N*_A_, *x*)/*n_i_*, where *X* = log_10_(*N*_A_/10^16^ cm^−3^. To obtain an acceptable analytic fit of ${Y_{p}}^{f}$ we want to satisfy for p-type Ga_1−_*_x_*Al*_x_*As the same conditions placed on the relative residual standard deviation and the number of fitting parameters for $Y_{p}^{f}$ as those given in Sec. 4 for $Y_{n}^{f}$.

## 6. Data Analysis and Final Results for *n*_ie_ in p-Type Ga_1−_*_x_*Al*_x_*As

We show in this Section that using a combination of separable functions, melding functions, and transformations on the discrete data points in Fig. 2, and non-linear regression analyses leads to two-dimensional, closed form analytic expressions for the normalized effective intrinsic carrier concentration at 300 K in p-type Ga_1−_*_x_*Al*_x_*As as a function of the mole fraction of AlAs *x* between 0.0 and 0.3 and the acceptor density *N*_4_ between 10^16^ cm^−3^ and 10^20^ cm^−3^. Throughout our analyses, we rely substantially on graphics and keep the number of fitting coefficients to a minimum, subject to the constraint that the residual standard deviation, *S*_res_(*Y^f^*), in the original units be as small as possible. For this case, *N* = 98 in Eq. (9).

We want to obtain the function ${Y_{p}}^{f}=f(X,x)$ (in the two-dimensional continuum space spanned by *X* = log(*N*_A_/10^16^ cm^−3^) and *x*. This bounded two-dimensional continuum is given in Fig. 2 with 0 ≤ *X* ≤ 4 and 0.00 ≤ *x* ≤ 0.30. As with fitted functions, extreme caution must be exercised in extrapolating beyond these *X* and *x* limits.

Figure 5 of Ref. [3] suggests that the Mott transition in p-type Ga_1−_*_x_*Al*_x_*As occurs somewhere near *N*_A_ = 2 × 10^18^ A cm^−3^ or *X* = *X*_M_ = 2.3. This is the region for which the bound states in the distorted densities of states due to high concentration effects are merging with continuum states. Determining the value of *X*_M_ from such distorted densities of states is not precise. The main point is that we expect it to be somewhere in the approximate region 2 ≤ *X*_M_ ≤ 3. Because of the uncertainty in *X*_M_ determined from examining distorted densities of states, we treat *X*_M_ in the following statistical analyses of the theoretical data as another fitting parameter with a fixed value. The scatter in the data for *Y*_p_(*X*, *x*) as shown in Fig. 2 sufficiently far away from the Mott transition is minimal and comparable to that in Ref. [4] for the minority electron mobilities. But, the scatter in the data for *Y*_p_(*X*, *x*) in the vicinity of the Mott transition presents an additional challenge in obtaining acceptable analytic fits for use in device simulators. The six possible reasons for this scatter are given above in Sec. 2.2.

We first perform a Lowess [6] smoothing procedure on the numerical data in Table 2 to determine a lower bound on $S_{\mathrm{res}}({Y_{p}}^{f})$ for any ${Y_{p}}^{f}$ with an acceptable number of fitting parameters. We find that $0.191<S_{\mathrm{res}}<{Y_{p}}^{f}$.

Because any given analytic fit to discrete data points is not unique, we present here two procedures that yield statistically similar results. By so doing, we hope to illustrate that there may be some fine structure in *n*_ie_ near the Mott transition. But, at this stage in interpreting experimental measurements and in using existing computers to solve complex equations, we simply do not know whether the fine structure in the second fitting procedure is physically correct or whether it is an artifact from the fitting procedure itself. The first procedure is based on a single, separable function to represent *Y*_p_(*X*, *x*) and the second procedure is based on two functions melded at *X*_M_ = 2.3 to represent *Y*_p_(*X*, *x*).

### 6.1 Single, Separable Function

Again, we consider the discrete two-dimensional space given by the 98 data points in Fig. 2 and in Table 2. Using the methodology in Ref. [4], we find that an acceptable fit, which meets the conditions on residual standard deviation and number of fitting parameters, is obtained by the following steps:

1. Chose the *x* = 0.0 GaAs data for *Y*_p_(*X*, 0) as the base or reference function and fit *Y*_p_(*X*, 0) to the quartic function *A*(*X*), where
  *A*(*X*) = *a*_0_ + *a*_1_ *X* + *a*_2_ *X*^2^ + *a*_3_ *X*^3^ + *a*_4_ *X*^4^.
2. Using the fitting parameters *a_i_* from step 1 now as fixed parameters, fit *Y*_p_(*X*, *x*) to the function ${Y_{p}}^{f}=(X,x)$, where
  $${Y_{p}}^{f}\left(X,x\right)=A\left(X\right)+B\left(X,x\right)$$ (11)
  and *B*(*X*, *x*) is the Lorentzian expression
  $$B\left(X,x\right)=\left(b_{0}+b_{1}\;x\right)/\left[1+{\left\{\left(X-c\right)/d\right\}}^{2}\right].$$ (12)

Step 2 gives values for the remaining four fitting parameters *b*_0_, *b*_1_, *c*, and *d*. In summary, Eq. (11) for ${Y_{p}}^{f}=(X,x)$ is the two-dimensional, closed form analytic expression for the data set *Y*_p_(*X*, *x*) containing 98 discrete data points. The nine fitting parameters for the normalized effective intrinsic carrier concentration for p-type Ga_1−_*_x_*Al*_x_*As, *Y*_p_ = *n*_ie_ (p-type; *N*_A_, *x*)/*n*_i_, from Eq. (11) are presented in Table 4a, where the acceptor density is *N*_A_. All of the fitting parameters are dimensionless. The residual standard deviation is *S*_res_(*Y*) = 0.205. The other expressions in Eq. (11) are:

$$\begin{array}{l} A\left(X\right)=a_{0}+a_{1}\;X+a_{2}\;X^{2}+a_{3}\;X^{3}+a_{4}\;X^{4},\\ B\left(X,x\right)=\left(b_{0}+b_{1}\;x\right)/\left[1+{\left\{\left(X-c\right)/d\right\}}^{2}\right],\;\text{and}\\ \;X=\log_{10}\left(N_{A}/10^{16}{\mathrm{cm}}^{-3}\right). \end{array}$$

As stated before, the analytic fit in Eq. (11) is valid only within the bounded space of 0 ≤ *X* ≤ 4 and 0 ≤ *x* ≤ 0.30, and it must not be used beyond this bounded two-dimensional space in which it is derived. The solid curves in Fig. 2 give the analytic fit from using Eq. (11).

### 6.2 Two Functions Melded Near the Mott Transition

We now consider the case of two functions melded near the Mott transition. As before, we apply the methodology in Ref. [4] to the discrete two-dimensional space given by the 98 data points in Fig. 2 and in Table 2. We find that another statistically acceptable fit is possible when two melded functions are used by the following steps:

1. Choose the *x* = 0.0 GaAs data for *Y*_p_(*X*, 0) as the base function and fit *Y*_p_(*X*, 0) for all *X ≤ X*_M_, where *X*_M_ = 2.3, to the Gaussian function ${Y_{p}}^{f}=(X,0)=F_{<}(X)$, for which *F*_<_(*X*) = *a*_1_+ *b*_1_exp[−0.5(*X* − *X*_1_)/*σ*_1_)^2^]. This gives beginning values for the five fitting parameters *a*_1_, *b*_1_, *X*_1_, *σ*_1_, and *X*_M_.
2. Choose the *x* = 0.0 GaAs data for *Y*_p_(*X*, 0) as the base function and fit *Y*_p_(*X*, 0) for all *X* > *X*_M_ to the Gaussian function ${Y_{p}}^{f}=(X,0)=F_{>}(X)$, for which *F*_>_(*X*) = *a*_2_ + *b*_2_exp[−0.5(*X* − *X*_2_)/*σ*_2_)^2^]. This gives beginning values for the four additional fitting parameters *a*_2_, *b*_2_, *X*_2_, and *σ*_2_.
3. Use the unit step function *w*(*X*) to meld or combine the two functions *F*_<_(*X*) and *F*_>_(*X*), where *w*(*X*) = 1 for *X* ≤ *X*_M_ and *w*(*X*) = 0 for *X* > *X*_M_. Then fit *Y*_p_(*X*, 0) for all *X* in the region 0.0 ≤ *X* ≤ 4.0 to the function *F*(*X*) where
  $$F\left(X\right)=w\left(X\right)F_{<}\left(X\right)+\left[1-w\left(X\right)\right]F_{>}\left(X\right).$$ (13)
4. And finally, using the nine fitting parameters from step 3 as beginning parameters, fit *Y*_p_(*X*, *x*) to the function ${Y_{p}}^{f}(X,x)$, where
  $${Y_{p}}^{f}\left(X,x\right)=F\left(X\right)+G\left(x\right)\;\text{and}$$ (14)
  $$G\left(x\right)=a_{0}+b_{0}\;x.$$ (15)

Step 4 gives values for the final 11 fitting parameters.

In summary, Eq. (14) for ${Y_{p}}^{f}(X,x)$ is the two-dimensional, closed form analytic expression for the data set *Y*_p_(*X*, *x*) containing 98 discrete data points. The 11 fitting parameters for the normalized effective intrinsic carrier concentration for p-type Ga_1−_*_x_*Al*_x_*As, *Y*_p_ = *n*_ie_ (p-type; *N*_A_, *x*)/*n*_i_ from Eq. (14), are given in Table 4b. Again, all of the fitting parameters are dimensionless. The residual standard deviation is $S_{\mathrm{res}}({Y_{p}}^{f})=0.198$.

The other expressions in Eq. (14) are:

$$\begin{array}{l} F\left(X\right)=a_{1}+b_{1}\exp\left[-0.5\left(X-X_{1}\right)/\sigma_{1}{)}^{2}\right]\;\text{when}\;X<X_{c},\\ F\left(X\right)=a_{2}+b_{2}\exp\left[-0.5\left(X-X_{2}\right)/\sigma_{2}{)}^{2}\right]\;\text{when}\;X\geq X_{c},\\ G\left(x\right)=a_{0}+b_{0}\;x,\;\text{and}\\ \;X=\log_{10}\left(N_{A}/10^{16}{\mathrm{cm}}^{-3}\right). \end{array}$$

The crossover or melding boundary is at *X* = *X*_c_ = *X*_M_ =2.3 or *N*_A_ = 2 × 10^18^ cm^−3^.

The analytic fit in Eq. (14) is valid only within the bounded space of 0 ≤ *X* ≤ 4 and 0 ≤ *x* ≤ 0.30, and it must not be used beyond this bounded two-dimensional space in which it is derived. Again, combining Eq. (14) with other transport models for mobilities, bandgaps, and effective intrinsic carrier concentrations that are derived from the interpretation of electrical measurements on the devices themselves may lead to incorrect descriptions of the electrical and optical behavior unless extra care is taken to be consistent.

**Fig. 3:**
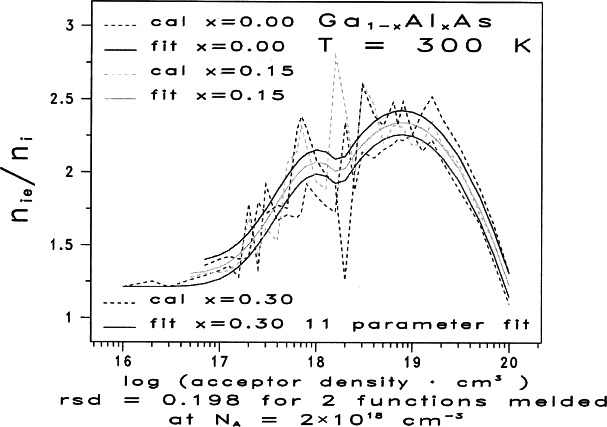
Normalized effective intrinsic carrier concentration ratios *n*_ie_/*n*_i_ for n-type Ga_1−_*_x_*Al*_x_*As as functions of the donor density for three representative values of mole fraction of AlAs. The three dashed curves show results from quantum mechanical calculations based on the Klauder fifth level of approximation for Ga_1−_*_x_*Al*_x_*As with *x* = 0.0, 0.15, and 0.30. The three solid curves show the closed-form, analytic fits based on Eqs. (14) and (15). H. S. Bennett, High Dopant and Carrier Concentration Effects in Gallium Aluminum Arsenide: Densities of States and Effective Intrinsic Carrier Concentrations, J. Appl. Phys. **83**, 3102 (1998).

The solid curves in Fig. 3 give the analytic fits from Eq. (14). We do not know whether the relative minima or fine structure at *X*_M_ in Fig. 3 is physically meaningful or is due to a partial fitting of the scatter in the data. More theoretical and experimental research will be needed to make a decision. Our main purpose in deriving Eq. (14) is to highlight that statistical analyses with a slightly smaller $S_{\mathrm{res}}({Y_{p}}^{f})$) suggests that structure may exist in *Y*_p_(*X*, *x*) near the Mott transition. But, until better computers and algorithms for calculating *n*_ie_ become available to reduce the numerical and computational scattering effects numbered 1 to 5 in Sec. 2.2 or until experiments verify the existence of such structure in *Y*_p_(*X*, *x*) near the Mott transition, we recommend for device simulation using only the analytic fit based on Eq. (11) and Table 4a.

## 7. Potential Significance of Results

Using the above Eq. (11) and applying additional results from calculations of mobilities in Ref. [7] to microwave HBTs [8] for linear power amplifiers may suggest different design strategies to optimize HBT performance. The calculated changes in carrier densities of states (DOS), band edges, band offsets, effective carrier concentrations *n*_ie_ and carrier mobilities due to high do-pant and carrier concentration effects in Ga_1−_*_x_*Al*_x_*As are given in Refs. [3] and [7] at 300 K for mole fractions *x* of AlAs between 0.0 and 0.3, for donor densities *N*_D_ between 10^16^ cm^−3^ and 10^19^ cm^−3^, and for acceptor densities *N*_A_ between 10^16^ cm^−3^ and 10^20^ cm^−3^. Only one quantum mechanical theory is used to treat both sides of the Mott transition in these calculations that give, with no fitting parameters to experimental measurements, an internally self-consistent description of carrier transport in Ga_1−_*_x_*Al*_x_*As/GaAs heterostructures for lasers, light emitting diodes, digital devices, and microwave devices. The predicted values for the distorted DOS, band edges, band offsets, *n*_ie_, and majority and minority mobilities differ from those values found in many simulations of Ga_1−_*_x_*Al*_x_*As/GaAs heterostructures. Many simulators set *n*_ie_/*n*_i_ = 1 in Ga_1−_*_x_*Al*_x_*As for all *N*_D_ or *N*_A_; approximate the minority electron mobility e (p-type; *N*_A_) with the majority electron mobility e (n-type; *N*_D_ = *N*_A_); and assert that all mobilities are monotonically decreasing functions of the dopant density. However, Fig. 5 in Ref. [7] shows that a relative minimum exists for *μ*_e_ (p-type; *N*_A_), and suggests that a different design strategy could be significant for linear HBT amplifiers in digital cellular phones. Because a relative minimum in the minority electron mobility as a function of the acceptor density exists, we have identified additional design considerations for HBT power amplifiers that would have not otherwise been known. The above relative minimum in the decade of 10^18^ cm^−3^ arises from dependencies of several competing scattering mechanisms on both the dopant and carrier densities. This relative minimum occurs because of the reduction of minority carrier (electron) scattering from plasmons associated with majority carriers (holes) and because of the removal of majority carriers (holes) from scattering the minority carriers (electrons) due to the Pauli exclusion principle for the majority carriers (holes).

If other parameters remain essentially the same as *N*_A_ increases from 6 × 10^18^ cm^−3^ to 6 × 10^19^ cm^−3^, then the following occurs:

1. the minority electron mobility [7] increases by a factor of 2.5,
2. the base transit time decreases by about a factor of 2.5, and
3. the base resistivity [9] decreases by about a factor of 10.

Combining these last three results into expressions from compact models [9] for microwave HBTs predicts increases in operating frequencies of about 40 % and in figures of merit (maximum frequencies at unity gain) of about 300 %. These estimates are considered to be upper limits because more rigorous simulations depend noticeably on both processing and operating parameters whose choices are determined by the application.

## 8. Conclusions

We have constructed two-dimensional, closed-form analytic functions for the normalized effective intrinsic carrier concentrations in Ga_1−_*_x_*Al*_x_*As at 300 K that are functions of dopant densities and mole fractions of AlAs. The results are important for device modeling because of the need to have accurate values for normalized effective intrinsic carrier concentrations, which in turn allow improved design of Ga_1−_*_x_*Al*_x_*As heterostructures used in telecommunications and optoelectronic systems; for example, digital cellular phones and modulators in optical communications systems.

The mobilities reported in Refs. [4] and [7] and the effective intrinsic carrier concentrations reported in this paper should be used together as a consistent set of input models for device simulators. Combining portions of the results in Ref. [4] or [7] and in this paper with other models for these quantities derived from the interpretation of electrical measurements on devices themselves requires care to make certain that the resulting descriptions are physically consistent.

The next tasks are to put these results into optoelectronic, microwave, and electronic device simulators; to determine the differences in predictions between the usual physical models used in simulators and the alternative physical models given is this paper; and to compare such predictions with measurements on devices of interest to companies and researchers. By so doing, more predictive simulations should be possible.

## Figures and Tables

**Table 1:** Two-dimensional array of data points from theoretical calculations^a^ of the normalized effective intrinsic carrier concentration for n-type Ga_1−_*_x_*Al*_x_*As, *Y*_n_ = *n*_ie_ (n-type; *N*_D_, *x*)/*n*_i_, where the acceptor density is *N*_D_, the mole fraction is *x*, and the intrinsic carrier concentration is *n*_i_

*N*_D_		*Y*_n_	
		*x*	
cm^−3^	0.00	0.15	0.30
1.00 × 10^16^	1.1100	1.1060	1.1180
2.00 × 10^16^	1.1350	1.1480	1.1470
3.00 ×10^16^	1.1620	1.1610	1.1880
5.00 × 10^16^	1.1730	1.1880	1.2240
7.00 ×10^16^	1.1950	1.2360	1.2440
1.00 × 10^17^	1.2420	1.2380	1.2670
2.00 × 10^17^	1.2380	1.2650	1.3390
3.00 × 10^17^	1.2630	1.3480	1.3420
5.00 × 10^17^	1.2720	1.3370	1.3650
7.00 × 10^17^	1.2100	1.2860	1.3710
1.00 × 10^18^	1.1520	1.2440	1.3480
2.00 × 10^18^	0.9447	1.1000	1.2470
3.00 × 10^18^	0.7523	0.9454	1.0940
5.00 × 0^18^	0.4682	0.6648	0.8556
7.00 × 10^18^	0.3062	0.4651	0.6374
1.00 × 10^19^	0.1558	0.2846	0.4373

a H. S. Bennett, High Dopant and Carrier Concentration Effects in Gallium Aluminum Arsenide: Densities of States and Effective Intrinsic Carrier Concentrations, J. Appl. Phys. **83**, 3102 (1998).

**Table 2:** Two-dimensional array of data points from theoretical calculations^a^ of the normalized effective intrinsic carrier concentration for p-type Ga_1−_*_x_*Al*_x_*As, *Y*_p_ = *n*_ie_ (p-type; *N*_A_, *x*)/*n*_i_, where the acceptor density is *N*_A_, the mole fraction is *x*, and the intrinsic carrier concentration is *n*_i_. The blank entries in this table means that the computer program did not converge to a solution after several hours

*N*_A_		*Y*_p_	
		*x*	
cm^3^	0.00	0.15	0.30
1.00 × 10^16^	1.2130		
2.00 ×10^16^	1.2520		
3.00 × 10^16^	1.2130		
5.00 × 10^16^	1.2630	1.2790	
7.00 ×10^16^	1.2920	1.3050	1.3610
1.00 ×10^17^	1.3270	1.3140	1.4030
1.26 × 10^17^	1.3590	1.3280	1.4250
1.58 × 10^17^	1.2810	1.3830	1.4060
2.00 ×10^17^	1.7860	1.6860	1.4130
2.51 ×10^17^	1.3410	1.4340	1.7980
3.00 × 10^17^	1.9170	1.6160	1.7170
3.98 × 10^17^	1.6700	1.5310	1.7780
5.00 × 10^17^	1.7110	2.0640	1.7560
6.31 × 10^17^	1.6900	2.1440	2.2950
7.00 × 10^17^	1.7230	2.3180	2.3850
7.94 × 10^17^	1.9230	2.1820	2.3010
1.00 × 10^18^	1.8410	1.9350	2.0880
1.26 × 10^18^	1.7740	1.8890	1.9840
1.58 × 10^18^	1.7290	2.7970	1.9220
2.00 × 10^18^	2.3430	2.4400	1.2630
2.51 × 10^18^	1.8800	1.9830	2.0300
3.00 × 10^18^	2.1420	2.6200	2.6050
3.98 × 10^18^	2.1010	2.4590	2.3890
5.00 × 10^18^	2.1570	2.2010	2.3240
6.31 × 10^18^	2.1970	2.3240	2.4780
7.00 ×10^18^	2.2300	2.3240	2.3290
7.94 × 10^18^	2.2210	2.3080	2.4870
1.00 × 10^19^	2.2640	2.1820	2.2660
1.26 × 10^19^	2.1490	2.2080	2.4210
1.58 × 10^19^	2.2250	2.3510	2.5230
2.00 × 10^19^	2.0840	2.2000	2.3770
3.00 × 10^19^	1.8870	2.0650	2.1870
5.00 × 10^19^	1.6470	1.7110	1.8870
7.00 × 10^19^	1.3820	1.5260	1.6390
1.00 × 10^20^	1.0990	1.2210	1.3160

a H. S. Bennett, High Dopant and Carrier Concentration Effects in Gallium Aluminum Arsenide: Densities of States and Effective Intrinsic Carrier Concentrations, J. Appl. Phys. **83**, 3102 (1998).

**Table 3:** The nine fitting parameters for the normalized effective intrinsic carrier concentration for n-type Ga_1−_*_x_*Al*_x_*As, *Y*_n_ = *n*_ie_ (n-type; *N*_D_, *x*)/*n*_i_ from Eq. (10), where the donor density is *N*_D_, the mole fraction of AlAs is *x*, and the intrinsic carrier concentration is *n*_i_.^a^ $Y_{n}^{f}(X,x)=\exp[A(X)+B(X,x)]$, where *A* (*X*) = *a*_0_ + *a*_1_ *X* + *a*_2_ *X*^2^ + *a*_3_ *X* ^3^ + *a*_4_ *X*^4^, and *B*(*X*, *x*) = (*b*_0_ + *b*_1_ *x*)/[1 + {(*X*−*c*)/*d*}^2^], and *X* = log_10_(*N_D_*/10^16^ cm^−3^). All of the fitting parameters are dimensionless. The ratio is the estimated value divided by the estimated standard deviation. The residual standard deviation is *S*_res_(*Y*) = 0.017

Reference function fitting parameters	Estimated value	Estimated standard deviation	Ratio
*a*_0_	0.143872	0.1445 × 10^−1^	10.
*a*_1_	0.977773 × 10^−1^	0.5080 × 10^−1^	1.9
*a*_2_	0.670024 × 10^−1^	0.1093	0.61
*a*_3_	0.213912 × 10^−3^	0.7608 × 10^−1^	0.28 × 10^2^
*a*_4_	−0.897642 × 10^−2^	0.1902 × 10^−1^	−0.47

Mole fraction function fitting parameters	Estimated value	Estimated standard deviation	Ratio
*b*_0_	−2.59105	0.3989	−6.5
*b*_1_	4.17080	0.5057	8.2
*c*	3.20507	0.4841 × 10^−1^	66.
*d*	0.448472	0.2598 × 10^−1^	17.

a H. S. Bennett, High Dopant and Carrier Concentration Effects in Gallium Aluminum Arsenide: Densities of States and Effective Intrinsic Carrier Concentrations, J. Appl. Phys. **83**, 3102 (1998).

**Table 4a:** The nine fitting parameters for the normalized effective intrinsic carrier concentration for p-type Ga_1−_*_x_*Al*_x_*As, *Y*_p_ = *n*_ie_ (p-type; *N*_A_, *x*)/*n*_i_ from Eq. (11), where the acceptor density is *N*_A_, the mole fraction of AlAs is *x*, and the intrinsic carrier concentration is *n*_i_.^a^ ${Y_{p}}^{f}(X,x)=A(X)+B(X,x)$, where *A* (*X*) = a_0_ *+ a*_1_ *X* + *a*_2_ *X*^2^ + a_3_ *X*^3^ +a_4_ *X*^4^, and B(*X*, *x*)*=(b*_0_ +b_1_ *x*)/[1*+*{*X*−)/*d*}^2^], and (*X*)= log_10_(*N*_A_/10 cm^−3^) All of the fitting parameters are dimensionless. The ratio is the estimated value divided by the estimated standard deviation. The residual standard deviation is *S*_res_(*Y*) = 0.205

Reference function fitting parameters	Estimated value	Estimated standard deviation	Ratio
*a*_0_	1.20595	0.1191	10.
*a*_1_	0.171915 × 10^−1^	0.3808	0.45× 10^−1^
*a*_2_	0.698161 × 10^−1^	0.3828	0.18
*a*_3_	0.107279	0.1424	0.75
*a*_4_	−0.319846 × 10^−1^	0.1747 × 10^−1^	−1.8

Mole fraction function fitting parameters	Estimated value	Estimated standard deviation	Ratio
*b*_0_	0.350316	0.8791 × 10^3^	0.40 × 10^−3^
*b*_1_	0.102178 × 10^2^	0.2561 × 10^5^	0.40 × 10^−3^
*c*	0.414650 × 10^7^	0.8665 × 10^10^	0.48 × 10^−3^
*d*	−0.100259 × 10^7^	0.3410 × 10^10^	−0.29× 10^−3^

a H. S. Bennett, High Dopant and Carrier Concentration Effects in Gallium Aluminum Arsenide: Densities of States and Effective Intrinsic Carrier Concentrations, J. Appl. Phys. **83**, 3102 (1998).

**Table 4b:** The 11 fitting parameters for the normalized effective intrinsic carrier concentration for p-type Ga_1−_*_x_*Al*_x_*As, *Y*_p_ = *n*_ie_ (p-type; *N*_A_, *x*)/*n*_i_ from Eq. (14), where the acceptor density is *N*_A_, the mole fraction of AlAs is *x*, and the intrinsic carrier concentration is *n*_i_.^a^ ${Y_{p}}^{f}(X,x)=F(X)+G(x)$, where *F* (*X*) = *a*_1_ + *b*_1_ exp[−0.5(*X* − *X*_1_)/*σ*_1_)^2^] when *X* ≥ *X*_c_, and where *F* (*X*) = *a*_2_ + *b*_2_ exp[−0.5(*X* − *X*_1_)/*σ*_2_)^2^] when *X* ≥ *X*_c_. The function *G* (*x*) = *a*_0_ + *b*_0_ *x* and *X* = log_10_(*N*_A_/10^16^ cm^−3^. The crossover or melding boundary is at *X* = *X*_c_ = *X*_M_ = 2.3 or *N*_A_ = 2 × 10^18^ cm^−3^. All of the fitting parameters are dimensionless. The ratio is the estimated value divided by the estimated standard deviation. The residual standard deviation is *S*_res_(*Y*) = 0.198

Reference function fitting parameters	Estimated value	Estimated standarddeviation	Ratio
*a*_1_	0.114993	1.415 × 10^3^	0.81 × 10^−4^
*b*_1_	0.776818	0.8468 × 10^−1^	9.2
*X*_1_	2.01657	0.8466 × 10^−1^	24.
*σ*_1_	0.440836	0.9605 × 10^−1^	4.6
*a*_2_	−8.67183 × 10^1^	3.362 × 10^3^	−0.26 × 10^−1^
*B*_2_	8.78811 × 10^1^	3.050 × 10^3^	0.29 × 10^−1^
*X*_2_	2.88904	0.4620 × 10^1^	63.
*σ*_2_	6.94881	1.229 × 10^2^	0.57 × 10^−1^

Mole fraction function fitting parameters	Estimated value	Estimated standarddeviation	Ratio
*a*_0_	1.09872	1.415 × 10^3^	0.78 × 10^−3^
*b*_0_	0.550019	0.1661	3.3

a H. S. Bennett, High Dopant and Carrier Concentration Effects in Gallium Aluminum Arsenide: Densities of States and Effective Intrinsic Carrier Concentrations, J. Appl. Phys. **83**, 3102 (1998).

## Footnotes

1 Certain commercial equipment, instruments, or materials are identified in this paper to foster understanding. Such identification does not imply recommendation by the National Institute of Standards and Technology, nor does it imply that the materials or equipment identified are necessarily the best available for the purpose.
